# A Novel Skin Closure Technique for the Management of Lacerations in Thin-Skinned Individuals

**DOI:** 10.7759/cureus.10702

**Published:** 2020-09-29

**Authors:** Kenneth Joyce, Shirley Potter

**Affiliations:** 1 Department of Plastic, Reconstructive, and Aesthetic Surgery, Mater Misericordiae Hospital, Dublin, IRL; 2 Department of Plastic, Reconstructive and Aesthetic Surgery, Mater Misericordiae Hospital, Dublin, IRL

**Keywords:** wound therapy, surgical glue

## Abstract

Suturing thin, fragile skin, particularly in elderly patients, is often problematic and presents a challenge to many clinicians. We describe a novel technique that re-enforces the edges of such thin fragile skin, with the use of topical skin adhesive, 2-octyl cyanoacrylate (Dermabond™; Ethicon, Somerville, NJ). This allows secure suture placement and application of tension to facilitate wound closure.

## Introduction

Epidermal and dermal atrophy, as well as decreased collagen content, are often a result of ageing and sun damage, which results in skin fragility. Such skin is prone to tearing and subsequent wound closure is often complicated by the suture cutting through the tissue [1]. Steri-Strips™ (3M, St. Paul, MN) alone often are not strong enough and, with increased wound tension, can place traction on the skin surface, resulting in blistering of the skin as the epidermis is sheared off [2]. Novel techniques have involved combining sutures and Steri-Strips™ to prevent ‘cheese-wiring’ of the skin [3]. Alternatives to Steri-Strips™ such as adhesive strips or polyethylene films have also been described [4,5]. One drawback of such techniques is the need to remove the sutures and adhesive strips which may further damage the fragile skin.

We describe a novel approach for the suturing of thin fragile skin. Suturing such skin, particularly in elderly patients, is often problematic and presents a challenge to many clinicians. Sutures tend to “cheese-wire” when even a minimum amount of tension is applied across the wound. We suggest a technique that re-enforces the edges of such thin fragile skin, allowing secure suture placement and application of tension to facilitate wound closure.

## Case presentation

**Figure 1:**
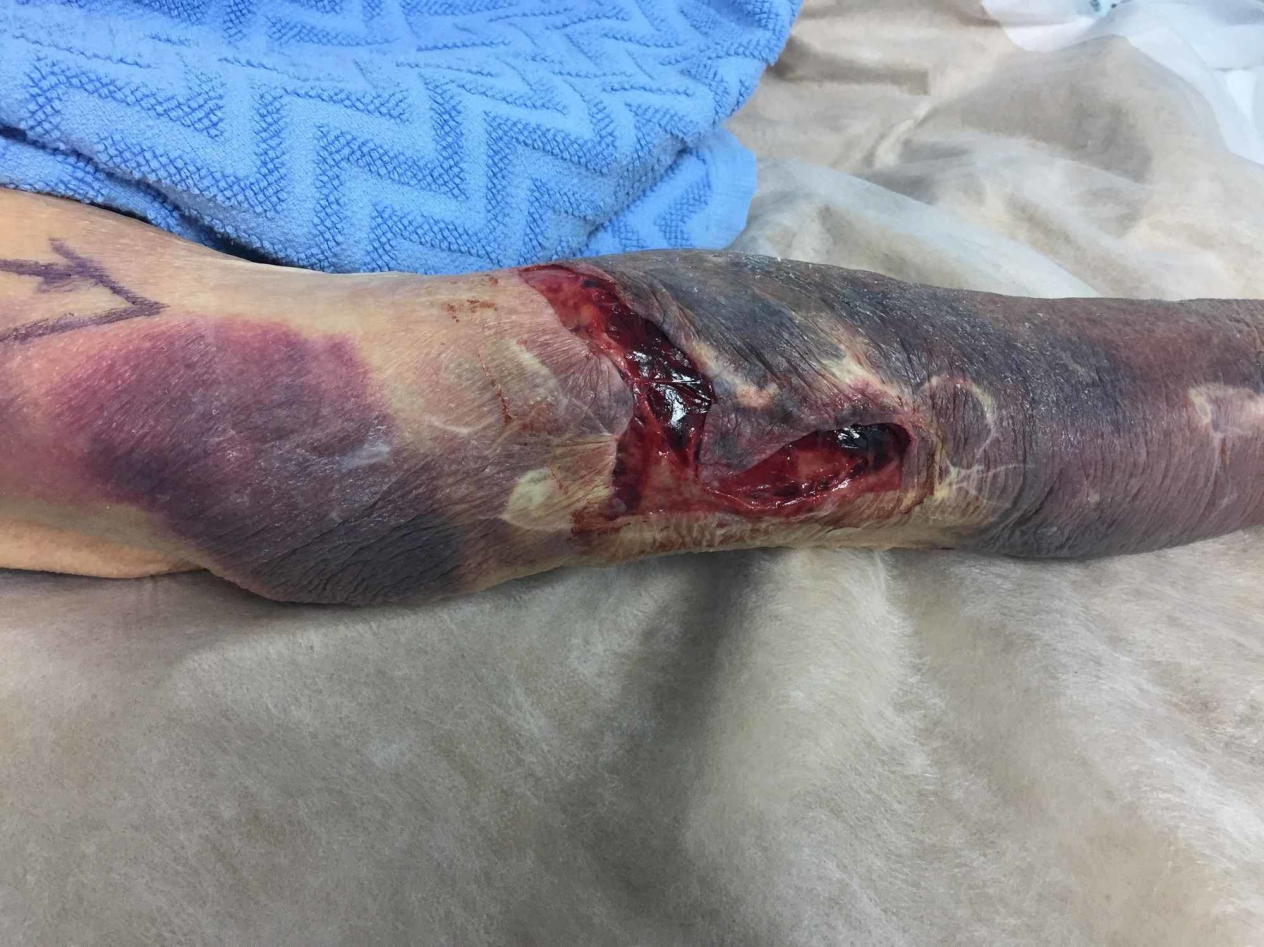
Laceration on dorsum of right forearm requiring suturing, with surrounding ecchymosis

We present the case of an 83-year-old lady who presented to our trauma clinic with a 18x3cm laceration to the dorsum of her right forearm (Figure 1). 

**Figure 2:**
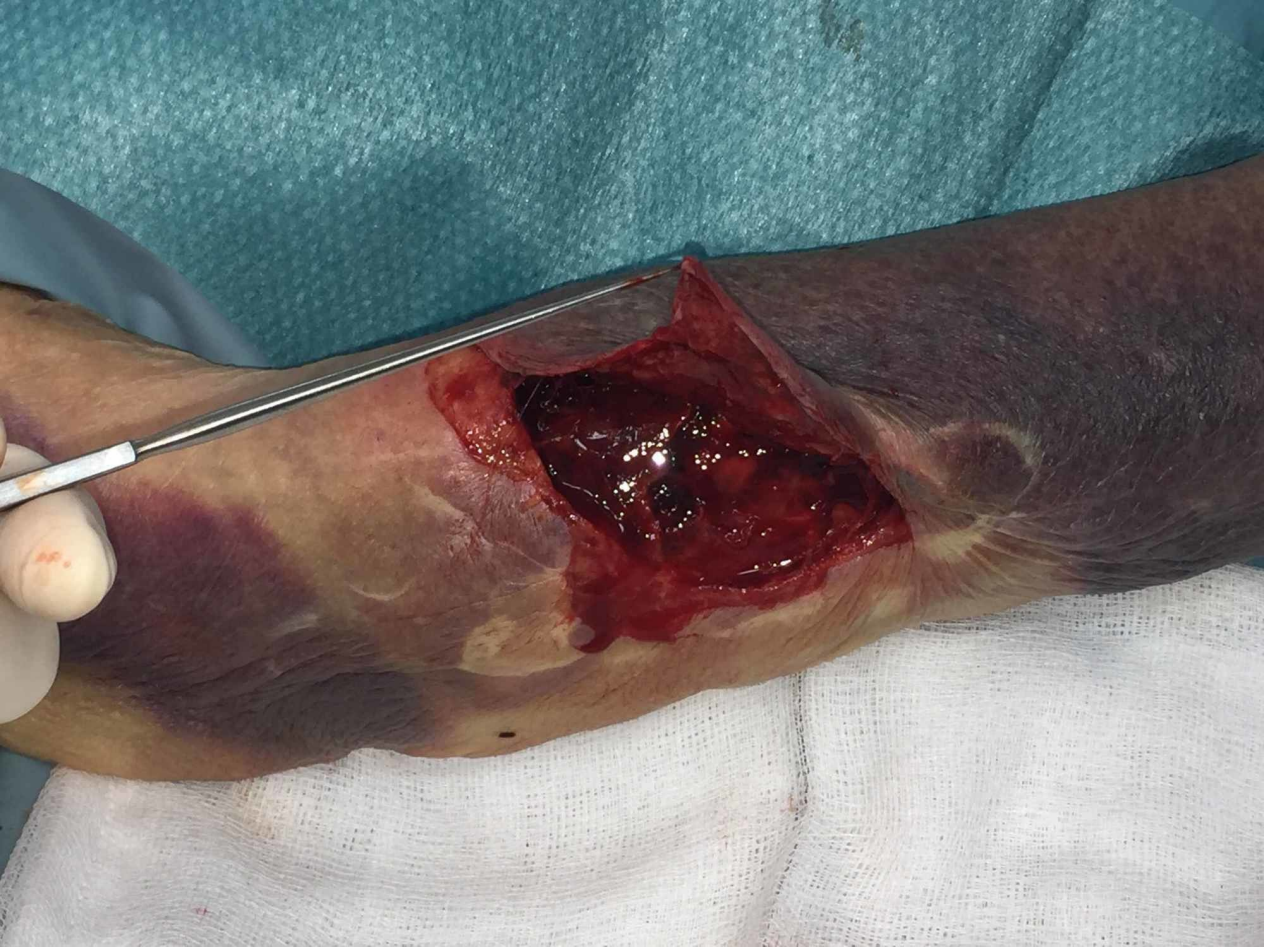
Degloving injury with dermal and epidermal atrophy

This occurred following a mechanical fall at home. The patient was transferred to the minor operating theatre for wound closure under local anaesthetic. The wound was irrigated prior to definitive closure. The degloving injury involved skin and subcutaneous fat. The underlying fascia was intact. Figure 2 demonstrates the thin fragile nature of the patient's skin due to age-related atrophy of epidermis and dermis.

**Figure 3:**
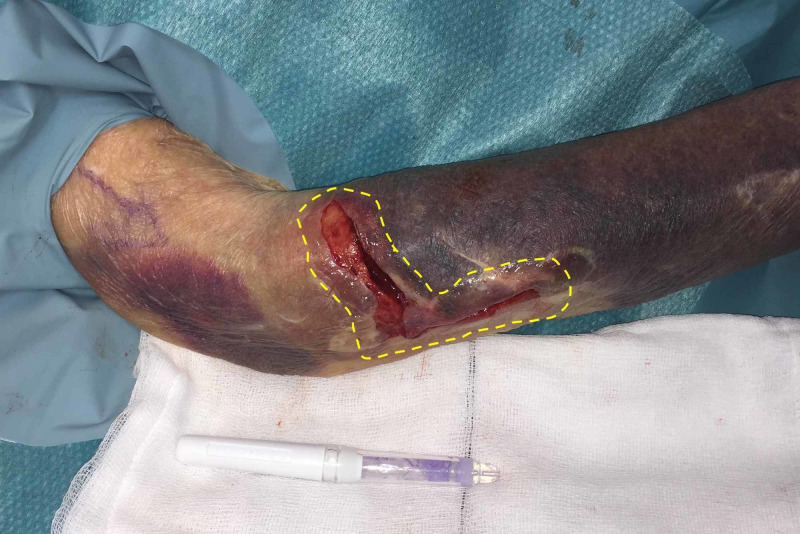
Yellow hatched line demonstrating area of skin adhesive application

The topical skin adhesive, 2-octyl cyanoacrylate (Dermabond™; Ethicon, Somerville, NJ), is applied around the perimeter of the wound, to increase strength and reinforce the wound edge (Figure 3).

**Figure 4:**
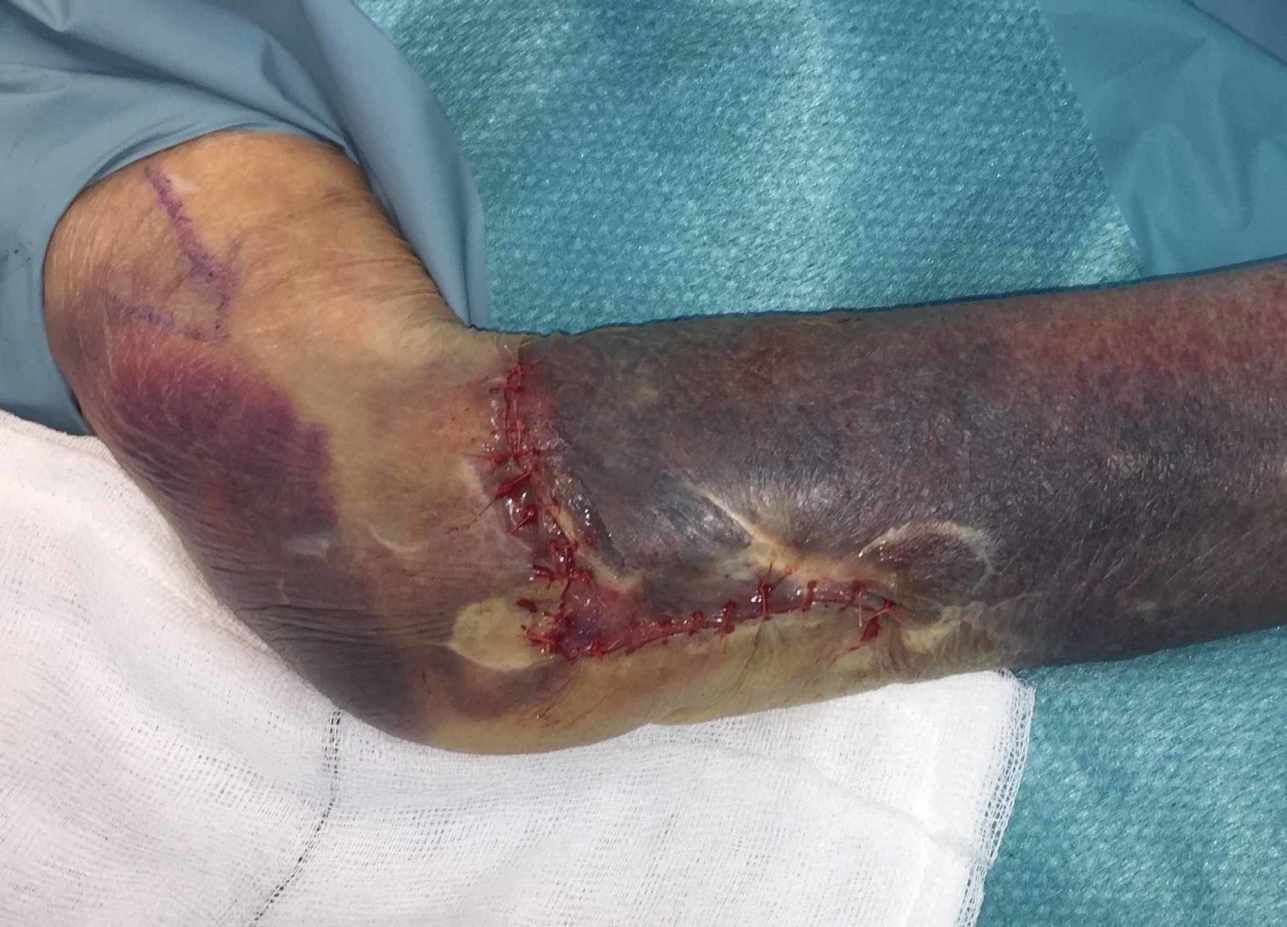
Wound sutured post-application of topical adhesive

Care must be taken to avoid allowing adhesive into the wound bed itself. The adhesive is allowed to dry completely (approximately two minutes). Sutures (simple or mattress) can then be placed through the adhesive/skin layer in one bite (Figure 4).

The skin adhesive reinforces the skin edge and prevents the suture from cutting through the skin. A significant amount of tension can therefore be applied to the suture to appose the wound edges and facilitate wound closure.

## Discussion

This technique allows full visualization of the skin edges, enabling the user to check for skin edge apposition along the length of the wound. The topical skin adhesive is absorbable (approximately five to seven days), and does not require removal. To date, we have not experienced any traction blistering and this technique does not interfere with wound healing. This simple and versatile novel technique is applicable to all parts of the body with thin or poor quality skin, helping to reduce complications and further morbidity.

## Conclusions

In conclusion, suturing of thin fragile skin is challenging, with sutures often pulling through the tissues. Use of topical skin adhesive, 2-octyl cyanoacrylate (Dermabond™) reinforces skin edges, thus allowing suture placement and wound closure.
